# Feasibility, Acceptability, and Preliminary Efficacy of Dignity Therapy in Patients With Early Stage Dementia and Their Family. A Pilot Randomized Controlled Trial

**DOI:** 10.3389/fpsyt.2021.795813

**Published:** 2021-12-24

**Authors:** Josef Jenewein, Hanspeter Moergeli, Tatjana Meyer-Heim, Peter Muijres, Irene Bopp-Kistler, Harvey M. Chochinov, Simon Peng-Keller

**Affiliations:** ^1^Department of Medical Psychology and Psychotherapy, Medical University of Graz, Graz, Austria; ^2^Department of Consultation-Liaison Psychiatry and Psychosomatic Medicine, University Hospital of Zurich, University of Zurich, Zurich, Switzerland; ^3^Waid City Hospital, University Geriatric Clinic, Zurich, Switzerland; ^4^Faculty of Theology, Spiritual Care, University of Zurich, Zurich, Switzerland; ^5^Cancer Care Manitoba Research Institute, University of Manitoba, Winnipeg, MB, Canada

**Keywords:** dementia, dignity, psychotherapy, psychological burden, family

## Abstract

**Purpose:** Dementia is the major cause for disability and dependence in older people and associated with considerable psychological burden. The aim of this study was to determine the feasibility, acceptability and preliminary efficacy of Dignity Therapy, a brief psychotherapeutic intervention to enhance dignity and reduce psychological burden, in patients with early stage dementia and in their families or close friends.

**Materials and methods:** In this randomized, waitinglist-controlled clinical trial a total of 54 patients with new diagnosis of early stage dementia and 54 study partners (spouses: *n* = 37; relatives: *n* = 14; close friends: *n* = 3) were randomly assigned to immediate treatment (*n* = 28) or delayed treatment (*n* = 26) after 3 months waiting. The main outcomes were feasibility: proportion of screened and invited patients who consented participation; Acceptability: number of drop-outs, and satisfaction with treatment; Efficacy: psychological burden (Hospital Anxiety and Depression Scale—HADS), quality of life (WHOQOL-Bref), and sense of dignity (Patient Dignity Inventory—PDI).

**Results:** In total 38.6% of all eligible patients (*n* = 140) consented and were enrolled. Along the study six participants (11.1%) dropped out. Patients' satisfaction with the treatment was high and with no significant difference between the groups. HADS scores were significantly lower in both groups at the 3-months follow-up (immediate group: mean difference = −2.69, SE = 0.85, *P* = 0.003; delayed group: mean difference = −1.97, SE = 0.89, *P* = 0.031). There was no significant group by time interaction effect (*F* = 0.71; df = 2, 70.3; *P* = 0.50). PDI scores only decreased significantly (i.e., improvement of dignity) in the immediate group (mean difference = −6.56, SE = 1.63, *P* < 0.001; delayed group: mean difference = −3.01, SE = 1.69, *P* = 0.081), but the group by time interaction effect was not statistically significant (*F* = 2.29; df = 1, 46.8; *P* = 0.14). Quality of life improved in some respects by the treatment, but the immediate and the delayed group did not differ significantly over time. After pooling patients' data of both groups, Dignity Therapy resulted in significant improvements in almost all outcome measures. Patients' family members/close friends reported high satisfaction with the intervention.

**Conclusions:** Our findings suggest that Dignity Therapy is feasible and highly accepted in patients with early stage dementia. Patients reported significant improvements, however, there was no significant effect of the intervention in the immediate treatment group compared to the delayed group.

## Introduction

Dementia is considered a global health concern, affecting over 50 million people worldwide and is estimated to increase to 131.5 million by 2050 (1). As dementia prevalence increases mainly with age, the number of individuals suffering from dementia will rise significantly in the future due to the aging population (2). Dementia is a clinical syndrome featuring a progressive decline in a variety of functions, including that of memory, language, and behavior (3). The cognitive deficits associated with dementia can interfere profoundly with daily activities and the management of everyday life, resulting in functional and social restrictions and a need for support in the completion of every day routine (2). Dementia, therefore, is one of the major causes for disability and dependence in older people (4). Additionally, in the absence of effective treatments, the diagnosis of dementia is frequently associated with considerable psychological burden in affected subjects as well as their family members. Dependence of care, physical and psychological impairments render individuals suffering from dementia vulnerable. Maintenance of dignity and quality of life by preserving physical and mental integrity, autonomy and social involvement are therefore key elements of care. Identifying methods that foster and retain the experience of dignity in patients living with dementia is of crucial importance.

Although patients with early stage dementia are not typically described as terminally ill, they are nevertheless, in their final phase of a consciously aware existence. While palliative and spiritual care are on the increase worldwide (5), less attention has so far been paid to the dignity-related concerns of elderly individuals who are not yet dying and are outside of palliative care settings (6). Being diagnosed with dementia can imply an incisive turning point in life, which might be associated with fear and loss of identity. Due to the high functional and social restrictions, patients with dementia frequently become dependent on support and therefore are at risk to lose autonomy and a sense of dignity. The loss of dignity in patients with early stage dementia is commonly associated with psychological and spiritual distress, despair, disability and loss of the will to live (7). These stressors might also affect well-being of partners and relatives. However, research on spiritual and dignity related issues for people with dementia and/or their partners is sparse. During the current demographic changes, dignified care in this context is becoming more urgent than ever. Enhancing the dignity of persons with dementia has been named as one of the WHO's top priorities (8, 9).

Dignity Therapy is a structured, individualized psychosocial intervention, developed to help patients with terminal illnesses to cope with the imminent end of their lives. Dignity Therapy was originally developed to reduce psychosocial and spiritual or existential distress in terminally ill cancer patients (10) by means of strengthening feelings of dignity, addressing existential and spiritual concerns, and preserving quality of life. Psychological and existential distress is addressed by discussing the themes most meaningful to patients and documenting their legacy in the form of a “generativity document.” “Generativity” refers to the notion that something meaningful, related to oneself, will survive, or transcend death. The “generativity document” may represent an attractive tool for patients with early stage dementia to share life experience, give insight into their life and express their own sense of self, meaning and purpose in life while they can, in the form of a lasting written legacy.

Research on Dignity Therapy in terminally-ill cancer patients started a decade ago and has since been conducted in several countries, including Canada (11), Australia (12), the United States (13), the United Kingdom (14), Denmark (15), Portugal (16), and Japan (17). Dignity Therapy has been shown to be highly feasible, to significantly improve quality of life, and to increase sense of dignity in patients with advanced cancer (10, 11, 15). Moreover, multiple studies reported a clear and consistently high rate of acceptance as well as high satisfaction and benefits including an enhanced sense of meaning and purpose among those who have experienced Dignity Therapy (18).

Dignity Therapy has also been carried out with patients suffering from a motor neuron disease (12), and with nursing home residents without cognitive impairment (6, 19). Furthermore, it has been tested in a feasibility study for people with early stage dementia demonstrating that Dignity Therapy is feasible, acceptable, and potentially effective for elderly people with dementia (20).

Like the terminally ill individuals, patients with early stage dementia often face existential and spiritual issues related to loss, disability, and death (6). Dignity Therapy, therefore, might also benefit patients with dementia (6, 20, 21). Related literature supports the assumption that Dignity Therapy may provide a powerful tool to mitigate end-of-life distress, maintain and heighten a sense of self, meaning and purpose as well as lessen suffering in patients with early stage dementia, prepare them for the future, and provide long term support to family members (6).

The aim of this study was firstly to determine the feasibility and acceptability of the intervention, and secondly—for the first time—to detect the preliminary efficacy of Dignity Therapy in patients with early stage dementia in a randomized, waitinglist-controlled clinical trial (RCT). The third aim of this study was to test whether Dignity Therapy might have a beneficial effect on family members (partner, relative) or close friend in terms of psychological burden and quality of life. Based on prior research (19), which applied Dignity Therapy to older people in care homes and which achieved a participation rate of 40%, we hypothesized that at least 40% of all eligible subjects would agree to participate, and secondly, that the drop-out rate would be lower than 25%. In terms of efficacy, we hypothesized a statistically significant improvement of psychological burden (HADS), sense of dignity (PDI), quality of life (WHOQOL-BREF), and spiritual well-being (FACIT-Sp-12).

## Materials and Methods

### Study Design and Participants

Because this study primarily focussed on feasibility and acceptability of Dignity Therapy in patients with early stage dementia, we chose a randomized, waiting list controlled design to achieve a large group of participants who received the intervention. The study was funded by Porticus foundation that did not have any role in the design or conduct of the study, analysis of the data, or preparation of the manuscript. The trial was registered at ClinicalTrials.gov (NCT03692988).

### Participants

Patients were recruited at the University Geriatric Outpatient-Center Waid, Switzerland, between March 2019 and March 2021. Patients (all of them outpatients) were eligible for participation in the trial if they were aged 18 years or older, had been diagnosed with early stage dementia according to the Clinical Dementia Rating instrument (CDR^®^ score between ≥0.5 and ≤ 1.5), language proficiency, and had a study partner (romantic partner, relative or close friend) willing to participate in the study. Family members, who had not necessarily to be an informal caregiver, were present during the intervention and were integrated into the therapeutic process. They were allowed to answer questions as well and to assist patients if required. All patients and family members provided written informed consent (IC). The study protocol was approved by the local ethics committee (BASEC-Nr. 2018-01097).

### Procedures

Physicians at the study site consecutively made patients, diagnosed with (very) mild dementia as indicated by a score on the Clinical Dementia Rating (CDR) (22) of ≥0.5 and ≤ 1.5, aware of the study and handed out study information for them to take home. CDR was obtained by experienced clinicians using a standard scoring algorithm based on scores in six domains indicating the severity of dementia (CDR 0 = cognitively normal, CDR 0.5 = very mild, CDR 1 = mild, CDR 2 = moderate, and CDR 3 = severe dementia).

**Figure 1:**
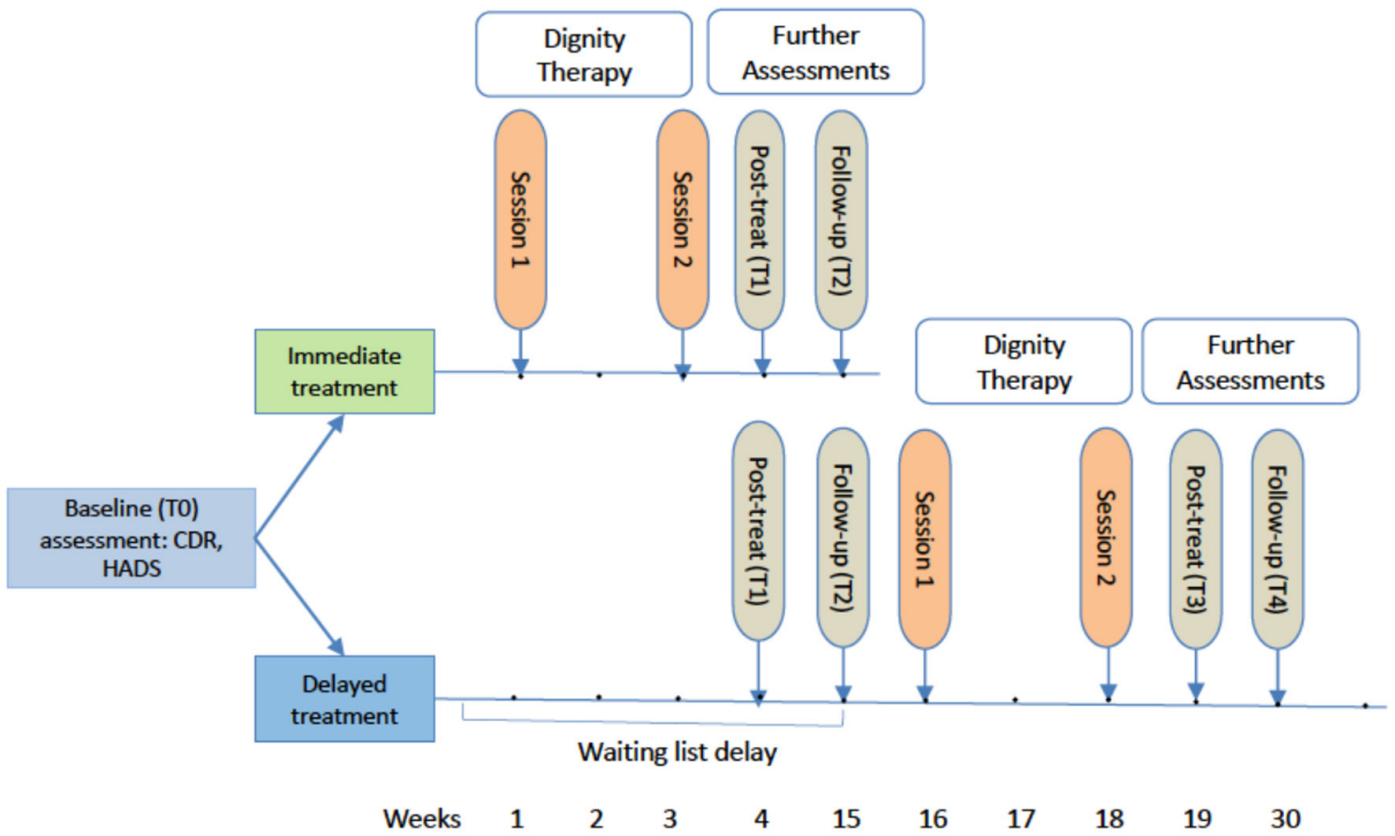
Study timeline from baseline assessment to the 1-week post-treatment and 3-months follow-up. T0, Baseline; T1, post-treatment; T2, 3-months follow-up; T3, post-treatment delayed group; T4, 3-months follow-up delayed group; CDR, Clinical Dementia Rating; HADS, Hospital Anxiety and Depression Scale.

The interested patients were then contacted by the study coordinator within a few days for a screening visit. In the screening visit the study coordinator reassured for accuracy of the inclusion/exclusion criteria and ensured that invited patients and family members were fully informed about the purpose, procedure, potential risks, and benefits of participating in the study, including not receiving any remuneration for study participation. After a further period of at least 24 h to consider and discuss their participation, the IC forms were dated and signed by both participants. Finally, participants were scheduled for the baseline assessment and thereafter randomly assigned to one of two study conditions (Figure 1). Randomization was performed by the data-management program secuTrial^®^ in a 1:1 ratio. Baseline assessments included questionnaires to measure psychological burden (Hospital Anxiety and Depression Scale -HADS), quality of life (WHOQOL-BREF), sense of dignity (Patient Dignity Inventory- PDI), and spiritual well-being (FACIT-Sp-12).

### Immediate Treatment Condition

The week after the baseline assessment (T0), the first Dignity Therapy session followed. The patient was accompanied by the study partner during the interview. The intervention was conducted by health care personnel trained in Dignity Therapy, who were neither involved in the study organization nor data management.

Dignity Therapy is conceptualized as multi-dimensional psychosocial intervention for patient-centered care, including 10 core questions that guide an interview. The questions invite a discussion of meaningful events, achievements, and social roles. The questions also encourage patients to express things that remained unsaid and pass on life's lessons and personal wishes to next of kin. For this study the core questions developed by Chochinov (10) were slightly adapted for the needs of the patients (see Supplementary Appendix). Dignity Therapy consists of two sessions: an interview session, including an initial framing interview (about 60′ in total), and a session to review the draft generativity document (30′). The interview session was audio-taped, transcribed verbatim, edited, and read out as a draft to the participants by the dignity therapist. The final generativity document, corrected and approved by participants, was sent to the participants by post.

After the second session, participants (patients and family members) again received the brief 14-item questionnaire designed to assess states of anxiety and depression (HADS), as well as an Dignity Therapy Evaluation Questionnaire (DTEQ) to assess satisfaction with the intervention (see Supplementary Appendix), which had to be returned within a week post-treatment (T1, post-treatment assessment). Meanwhile, the edited, final version of the generativity document was produced, attractively packaged, and sent to the participant by post. About 3 months (week 15) after randomization (day 0), the participants were contacted again with the request to fill out the follow-up questionnaire (T2) and the DTEQ. The follow-up questionnaire was identical to the baseline questionnaire, although demographic questions were excluded.

### Delayed Treatment Condition

Patients in the delayed treatment group completed the standard assessments (T0, T1, and T2), but received no Dignity Therapy in addition to counseling provided as usual. They constituted a control condition enabling the comparison of data with the intervention group. After the waiting period and a completed follow-up assessment (T2, week 15) the delayed treatment group participants received the same intervention as the immediate group. The follow-up assessment in this group simultaneously served as the baseline assessment of the delayed intervention. To also determine the effect of the Dignity intervention, participants of the delayed condition were asked to once more complete a post-treatment (T3) in week 19, and a follow-up assessment (T4) 3 months (week 30) after the intervention.

Family members/close friends of both groups also completed assessments at T0 to T4, however they did not receive the PDI and the FACIT-Sp-12.

### Study Outcomes

The primary outcome of this trial was the feasibility and acceptance of Dignity Therapy provided to a sample of patients with early stage dementia and their partners/relatives in Switzerland. Feasibility was defined by the number of participants who signed ICs in proportion to the number of eligible subjects who were invited to participate in the study. Because this was the first time to apply this intervention in this specific clinical population, we considered Dignity Therapy as feasible if at least 40% of all eligible subjects consented. Acceptability was defined as the number of drop-outs occurring after enrollment and signing the IC. If the drop-out rate was 25% at most, we considered Dignity Therapy as well-accepted. Furthermore, to assess an additional aspect of acceptance we developed a nine items (Likert scale 1–5) comprising questionnaire for overall treatment satisfaction (DTEQ) with a minimum score of nine and maximum score of 45. In such a range a mean score of 36 (quite a bit satisfied) can be regarded as typical in health care (23). Internal consistency of the DTEQ was high at post-treatment as well as at follow-up (Cronbach's alpha = 0.92 and 0.87, respectively).

For the secondary outcomes we used standardized and validated self-report measures to determine the efficacy of Dignity Therapy in the immediate treatment group compared to the delayed condition in terms of psychological burden (HADS), sense of dignity (PDI), quality of life (WHOQOL-BREF), and spiritual well-being (FACIT-Sp-12). PDI and FACIT-Sp-12 were used only in patients and not in family members. All questionnaires have been used in comparable samples in prior research with limited (HADS) (24) to very good validity [PDI (25), WHOQOL-BREF (26), FACIT-Sp-12) (27).

### Measurements

The Hospital Anxiety and Depression Scale (HADS) is a 14-item questionnaire that was originally developed for use with patients in non-psychiatric hospitals (28). It is a validated and widely-used self-report measure that assesses individuals' self-perceived levels of depression and anxiety (29). It can be used to identify patients with elevated levels of symptoms that may be clinically relevant (probable anxiety: HADS anxiety score >7/HADS; probably depression: HADS depression score >7) (30). At baseline, Cronbach's alpha for the HADS total score was 0.87 and 0.82 at follow-up (T2/T4).

The Patient Dignity Inventory - German Version (PDI-G) is a valid and reliable 25-item questionnaire that specifically assesses dignity-related issues and identifies end-of-life dignity-related distress (31). The questionnaire was recently translated into German by Sautier et al. (32) and includes the factors: physical symptom distress, loss of sense of worth and meaning, loss of autonomy, anxiety, and uncertainty. We used only total scores ranging from 25 (not at all) to 125 (very much) in this study. Cronbach's alpha for the PDI total score was 0.95 at baseline (T0) and 0.92 at follow-up (T2/T4).

WHO Quality of Life Questionnaire (WHOQOL-BREF): The quality of life of partners/family members was assessed with the WHOQOL-BREF, which is not specific to any illness. The 26-item questionnaire encompasses the domains of physical and psychological health, social relationships, and environment, as well as overall quality of life and general health (33). At baseline, Cronbach's alpha ranged from 0.58 (social domain) to 0.81 (psychological domain) and from 0.48 (social domain) to 0.83 (psychological domain) at follow-up (T2/T4).

Functional Assessment of Chronic Illness Therapy Spiritual Wellbeing Scale (FACIT-Sp-12): The FACIT-Sp-12 (34) is a short, validated tool that specifically assesses spiritual components of quality of life. Response options include a five-point Likert scale ranging from 0 (not at all) to 4 (very much). The total spiritual well-being score ranges from 0 to 48. Cronbach's alpha for the FACIT-Sp-12 total score was 0.82 at baseline (T0) and 0.81 at follow-up (T2/T4).

### Sample Size Calculation

To determine the sample size in advance a power analysis was performed considering hypotheses regarding feasibility, acceptance, and treatment satisfaction in the whole sample of patients as well as symptom reduction in the RCT condition. About feasibility a minimum of 38 screened patients would have to be invited for participation to conclude that an expected preferable rate of 60% consenting patients is significantly higher than a minimal rate of 40% with a power = 0.8 (type I error = 0.05, one-sided). As to acceptability we expected a drop-out rate after informed consent of 10%. Should the drop-out rate be 25% or more, Dignity Therapy would be considered as badly accepted. With a minimum of 42 patients entered the study (signed informed consent) it would be possible to conclude that the expected drop-out rate is significantly lower than 25% with 80% power (type I error = 0.05, one-sided). Additionally, we expected the overall satisfaction with Dignity Therapy not to be significantly lower than a reference value (reference = 36, SD = 6.0, scale ranging from 9 to 45). At most, the difference should correspond to an effect size of Cohen's d = 0.5. For the given effect size with a power of 80% and a type I error = 0.05 (one-sided), a total sample size of 27 patients would be required. Presuming a moderate symptom reduction (HADS) an effect size of Cohen's d = 0.5 in the intervention group, compared to no effect in the waiting list group, a sample size of 64 patients per group (128 patients in total) would be needed to generate a significant interaction effect between group and time with a power = 0.8 and type I error = 0.05. Given that the waiting list controlled design was secondary and the sample size calculations further above, we reduced the sample size to 24 patients per group (48 patients in total) which halved the power to 40% in the RCT condition. However, in a combined group consisting of both arms (single-armed intervention study), a significant decrease in symptom load after intervention could be determined with a power of 92% (expected effect: Cohen's d = 0.5, type I error = 0.05). Because we expected 10% of participants to drop out, recruiting 54 patients in total (27 per group) was necessary. Power and sample-size analyses were calculated with Stata 16.1 (one-sample proportion test, one-sample mean test, repeated-measures analysis of variance).

### Statistical Analyses

Descriptive statistics of demographic, clinical and psychological variables were calculated with mean, standard deviation (SD), and percentage (%) as appropriate. To calculate the 95% confidence intervals (CI_95%_) for the proportions of patients consenting to participate (feasibility) and dropping out after informed consent (acceptance) bootstrapping based on 1,000 bootstrap samples was performed. Overall satisfaction with Dignity Therapy was tested against the expected reference score using one-sample *t*-test.

Under the condition of a randomized controlled trial (RCT) linear mixed model analyses for all available outcome data were used to test the effect of group (i.e., immediate vs. delayed condition), time (baseline, post-treatment, 3-months follow-up) and group^*^time interaction on outcome measures. Based on estimated marginal means *post-hoc* comparisons of time points within the groups as well as comparisons of groups at different times were performed. The interaction effect between group and time was relevant to evaluate for different improvement depending on group membership. Performing linear mixed model analyses with all available outcome data corresponds to a consideration of all patients with intention to be treated at baseline (intention-to-treat-analyses: ITT). For further analyses the data from both groups were combined by merging the T2, T3, and T4 assessments of the delayed treatment group with the T0, T1, and T2 assessments of the immediate treatment group. The same procedure was applied to the data from the study partners, and it resulted in a data set including the whole sample of patients and study partners with assessments before and after treatment. Again, linear mixed model analyses for all available outcome data were used including *post-hoc* comparisons based on estimated marginal means. The Statistical Package for the Social Sciences (SPSS, version 26) was used to perform the statistical analyses, with a probability value of 0.05 considered to be statistically significant.

## Results

### Feasibility, Acceptability, and Overall Treatment Satisfaction

**Figure 2:**
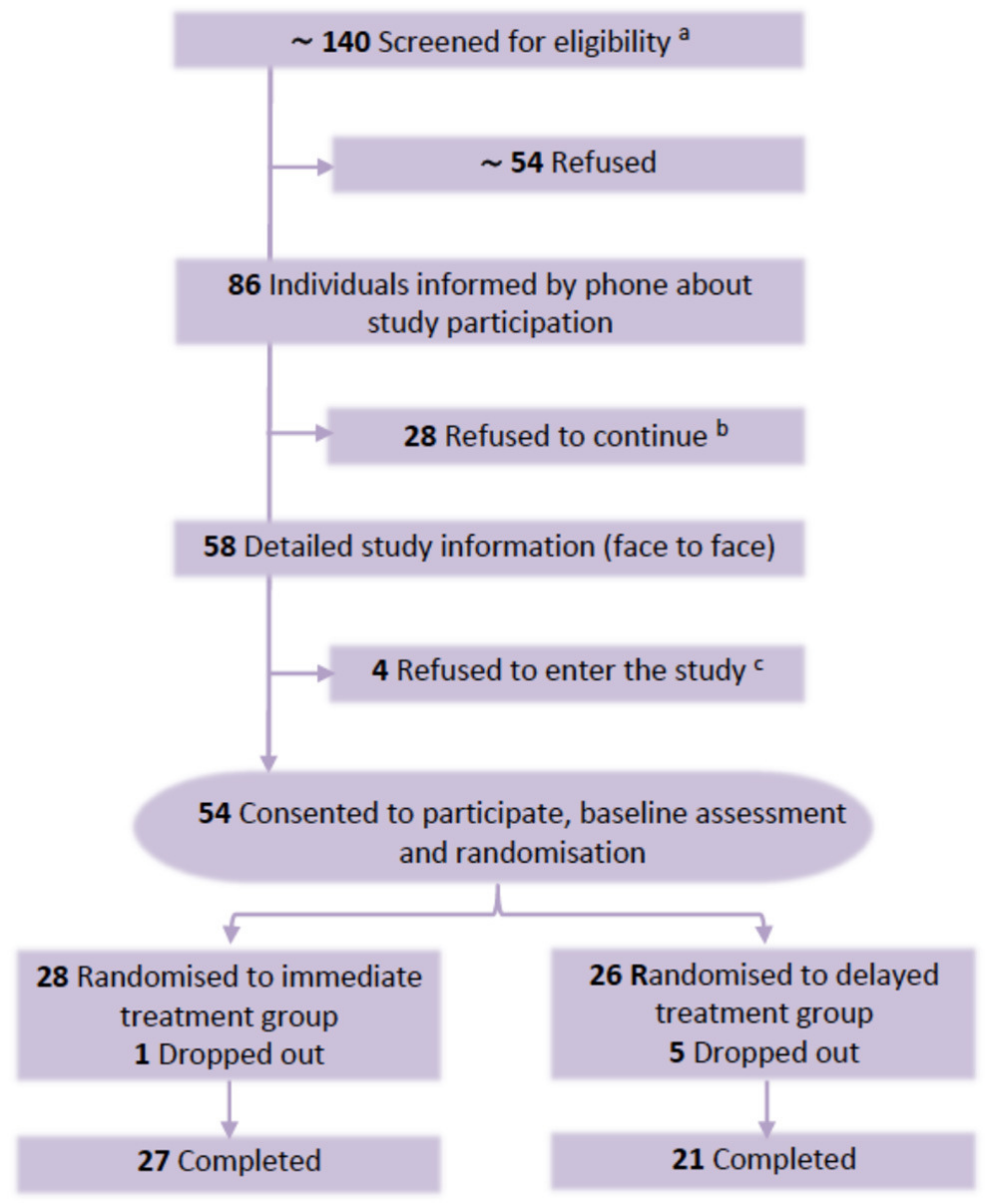
CONSORT diagram of participant flow. ^a^Due to changes in study personnel and pandemic problems the number of screened patients had to be estimated retrospectively from exact numbers of the main recruiting physician. ^b^Refused after detailed information by phone about study. Reasons for refusal: study requirements such as filling in questionnaires (*n* = 18); not wanting the intervention (*n* = 6); mental or physical problems (*n* = 4). ^c^Refused after detailed face to face information: study requirements (n=2); mental or physical problems (*n* = 2).

Between March 2019 and March 2021, a total of 86 eligible patients were registered and afterwards contacted by the study coordinator. Due to some changes in the team of recruiting physicians and a recruiting stop from March 2020 to May 2020 because of the pandemic, we had to retrospectively estimate the exact number of invited participants. The main recruiting physician invited a total of 65 suitable patients, of whom 40 (61.5%) agreed to participate (i.e., willingness to be contacted by the study coordinator for full information). To estimate all eligible participants, we used the exact numbers of the main recruiting physician who invited a total of 65 eligible patients, of whom 40 (61.5%) agreed to participate. Based on the final registered participants (*N* = 86) and the recruiting rate of the main recruiting physician (61.5%) we estimated a total of 140 (86/61.5 × 100 = 139.8) eligible patients for all recruiting physicians (Figure 2). Of the finally registered 86 patients 54 (62.8%) consented and were enrolled after full face to face information about the study. Combining the recruiting rate and the consenting rate after full information a final participation rate of 38.6% (CI_95%_: 30.7–47.1%) was calculated. Main reasons for refusal after full information were related to study requirements such as questionnaires, appointments etc. (*n* = 20), not wanting the intervention (*n* = 6), and physical or mental problems (*n* = 6). At the beginning of the first lock-down on March 16th, 2020, already 42 of the intended 54 participants were included. During the lock-down, recruiting was fully stopped and scheduled face-to-face interventions were postponed, which was not associated with a higher drop-out rate (all drop-outs occurred before the lock-down). After the lock-down recruiting and interventions were continued using the same procedure as before. However, recruiting was significantly slowed because of the new organizational requirements at the hospital. Overall, we did not ascertain a lower acceptance of the invited patients after the begin of the pandemic.

Table 1 shows the sociodemographic characteristics of the 54 patients, among whom 28 (51.9%) were women and 26 (48.1%) men, with a mean (SD) age of 79.6 (6.3) years. We found no significant differences in sociodemographic characteristics between both treatment groups. Sociodemographic characteristics of study partners (spouses: *n* =37; relatives: *n* = 14; close friends: *n* = 3) are also reported in Table 1.

**Table 1:** Demographic and clinical characteristics of subjects with early stage dementia (*N* = 54) and family members/close friends (*N* = 54).

	**Patients (*****N*** **=** **54)**					**Family/Friends (*****N*** **=** **54)**	
	**Immediate group (*****n*** **=** **28)**		**Delayed group (*****n*** **=** **26)**		**Statistics**	**Both groups**	
**Variable**	**Mean (Min-Max)**	**SD**	**Mean (Min-Max)**	**SD**	***P* ^a^**	**Mean (Min-Max)**	**SD**
Age (years)	81.2 (70–91)	5.7	77.8 (63–93)	6.7	0.051	70.2 (39–90)	12.9
CDR^b^	0.92 (0.5–1.5)	0.26	0.96 (0.5–1.5)	0.25	0.244	n.a.	n.a.
	***N***	**%**	***N***	**%**		***N***	**%**
**Gender**							
Female	16	57.1	12	46.2		34	63.0
Male	12	42.9	14	53.8	0.586	20	37.0
**Marital status**							
Married	16	57.1	20	76.9		45	83.3
Cohabitation	1	3.6	0	0.0		1	1.9
Divorced	3	10.7	1	3.8		1	1.9
Widowed	8	28.6	3	11.5		2	3.7
Single	0	0.0	2	7.7	0.138	5	9.3
**Education**							
Obligatory	4	14.3	3	11.5		4	7.4
Apprenticeship	10	35.7	10	38.5		30	55.6
High school	5	17.9	2	7.7		1	1.9
College/university	9	32.1	11	42.3	0.729	18	33.3
**Employment status**							
Full	0	0.0	0	0.0		14	25.9
Part	4	14.3	1	3.8		19	35.2
Retired	24	85.7	25	96.2	0.353	21	38.9
**Religion**							
Catholic	10	35.7	7	26.9		8	14.8
Reformed	12	42.9	12	46.2		30	55.6
Other Christian	0	0.0	1	3.8		1	1.9
No confession	5	17.9	5	19.2		13	24.1
Other	1	3.6	1	3.8	0.920	2	3.7

a *Comparison between immediate and delayed group: Fisher's exact test or t-test when appropriate*.

b *Clinical Dementia Rating*.

Along the study six participants (11.1%; CI_95%_: 3.7–20.4%) dropped out, one in the immediate (before the intervention, felt overstrained by questionnaires) and five in the delayed treatment group (all before intervention: one patient died, three felt overstrained by study requirements, one reconsidered participation).

Overall treatment satisfaction, assessed by our nine items comprising questionnaire (DTEQ) with a minimum score of nine and maximum score of 45, was high (**Table 3**). Patients reported a mean satisfaction after the intervention of 37.8 (CI_95%_: 35.7–39.8) and 40.4 (CI_95%_: 39.1–41.6) at the 3-months follow-up. Thus, it was near or above the expected reference score (36.0). Study partners reported a similar satisfaction post-treatment of 36.3 (CI_95%_: 34.4–38.2) but were somewhat less satisfied at the 3-months follow-up (34.9; CI_95%_: 33.0–36.9).

### Treatment Outcome in RCT Condition

Results for all secondary outcomes are presented in Table 2. In the immediate treatment group, we found a statistically significant reduction of the HADS scores (Mean difference = −2.69, SE = 0.85, *P* = 0.003) as well as the PDI scores (Mean difference = −6.56, SE = 1.63, *P* < 0.001) at the 3-months follow-up. We further found a significant improvement in the physical, social, and environment domains of quality of life as well as in spiritual well-being. *Post-hoc* analyses also revealed a significant reduction of HADS scores in the delayed condition group (Mean difference = −1.97, SE = 0.89, *P* = 0.031), but no significant differences in all other measures. However, and maybe because of the limited power of the study, we found no statistically significant group by time interaction effect on all outcome measures (e.g., HADS: *F* = 0.71; df = 2, 70.3; *P* = 0.50; PDI: *F* = 2.29; df = 1, 46.8; *P* = 0.14; Qol-social: *F* = 2.39; df = 1, 49.7; *P* = 0.13; FACIT-Sp-12: *F* = 1.41; df = 1, 49.0; *P* = 0.24). The effect size for HADS scores (calculated in per protocol patients) at the 3-months follow-up was small (Cohen's d = 0.1; between groups effect corrected for baseline scores).

**Table 2:** Secondary outcomes in RCT condition (immediate treatment compared to delayed treatment group).

**Participant subgroup**	**Measures**	**T0** **Mean (SE)**	**T1** **Mean (SE)**	**T2** **Mean (SE)**	**Comparison (T2**–**T0)**		**Statistics**
					**Mean difference (SE)**	**95% CI**	***P*-value**
Immediate group (*n* = 28)	HADS total	8.39 (1.15)	8.19 (1.30)	5.70 (0.92)	−2.69 (0.85)	−4.40 to −0.99	0.003
	PDI	38.1 (2.43)	–	31.5 (2.47)	−6.56 (1.63)	−9.83 to −3.29	<0.001
	Qol-physical	79.1 (2.47)	–	84.2 (2.49)	5.14 (1.82)	1.48 to 8.81	0.007
	Qol-psychological	74.7 (2.90)	–	78.3 (2.92)	3.61 (1.88)	−0.16 to 7.39	0.060
	Qol-social	75.6 (2.82)	–	80.8 (2.82)	5.27 (2.55)	0.14 to 10.4	0.044
	Qol-environment	83.5 (2.20)	–	87.8 (2.22)	4.31 (1.77)	0.76 to 7.85	0.018
	Qol-overall	77.2 (3.33)	–	79.2 (3.37)	1.96 (2.98)	−4.03 to 7.95	0.514
	FACIT-Sp-12	33.6 (1.40)	–	36.4 (1.42)	2.74 (1.33)	0.06 to 5.42	0.045
Delayed group (*n* = 26)	HADS total	8.69 (1.19)	10.08 (1.33)	6.72 (0.96)	−1.97 (0.89)	−3.76 to −0.19	0.031
	PDI	37.9 (2.52)	–	34.9 (2.56)	−3.01 (1.69)	−6.41 to 0.39	0.081
	Qol-physical	78.3 (2.57)	–	79.2 (2.61)	0.88 (1.93)	−3.00 to 4.76	0.651
	Qol-psychological	76.0 (3.01)	–	76.0 (3.06)	0.01 (1.99)	−3.98 to 4.01	0.994
	Qol-social	79.8 (2.90)	–	79.4 (2.97)	−0.43 (2.66)	−5.76 to 4.91	0.873
	Qol-environment	84.3 (2.28)	–	86.7 (2.33)	2.44 (1.87)	−1.31 to 6.19	0.198
	Qol-overall	70.7 (3.46)	–	72.9 (3.55)	2.26 (3.15)	−4.06 to 8.58	0.475
	FACIT-Sp-12	34.5 (1.46)	–	35.0 (1.50)	0.44 (1.41)	−2.39 to 3.27	0.757

*T0, Baseline; T1, Post-treatment; T2, 3-months follow-up; Mean, Model Estimated Mean; SE, Standard Error of Mean*.

*HADS, Hospital Anxiety and Depression Scale; Qol, WHOQOL-Bref; PDI, Patient Dignity Inventory; FACIT-Sp-12, FACIT – Spiritual Well-Being*.

Interaction effects in all measures (group^*^time) were not significant, as were all between group effects at T0.

### Treatment Outcome in the Combined Sample

To further analyze the effect of the intervention the data from both groups were combined by merging the T2, T3, and T4 assessments of the delayed treatment group with the T0, T1, and T2 assessments of the immediate treatment group. The results are displayed in Table 3. We found significant differences between the baseline and 3-months follow-up scores in all measures, except Qol-overall.

**Table 3:** Treatment satisfaction and secondary outcomes in the whole sample of patients complemented by a comparison in study partners.

**Participant subgroup**	**Measures**	**T0** **Mean (SE)**	**T1** **Mean (SE)**	**T2** **Mean (SE)**	**Comparison (T2**–**T0)**^**d**^		**Statistics**
					**Mean difference (SE)**	**95% CI**	***P*-value**
Combined patient sample^a^ (*n* = 52)^b^	Treatment satisfaction (*n* = 48)^c^	–	37.8 (1.00)	40.4 (0.62)	2.60 (0.94)	4.49 to 0.72	0.008
	HADS total	7.62 (0.74)	8.74 (0.83)	6.15 (0.82)	−1.46 (0.70)	−2.84 to −0.08	0.039
	PDI	36.9 (1.58)	–	32.3 (1.61)	−4.63 (1.06)	−6.77 to −2.50	<0.001
	Qol-physical	78.9 (1.90)	–	82.2 (1.76)	3.25 (1.25)	0.73 to 5.77	0.013
	Qol-psychological	75.0 (2.12)	–	78.9 (1.97)	3.94 (1.64)	0.67 to 7.20	0.019
	Qol-social	77.0 (1.89)	–	81.2 (2.15)	4.11 (1.57)	0.97 to 7.25	0.011
	Qol-environment	84.8 (1.46)	–	88.8 (1.41)	3.99 (1.34)	1.33 to 6.66	0.004
	Qol-overall	75.0 (2.43)	–	78.6 (2.34)	3.62 (2.25)	−0.85 to 8.09	0.111
	FACIT-Sp-12	34.0 (0.98)	–	35.8 (1.00)	1.77 (0.87)	0.01 to 3.52	0.048
Study partners (*n* = 52)^b^	Treatment satisfaction (*n* = 48)^c^	–	36.3 (0.96)	34.9 (0.97)	−1.38 (0.62)	−2.64 to −0.13	0.032
	HADS total	9.87 (0.87)	9.21 (0.89)	9.69 (0.89)	−0.17 (0.67)	−1.50 to 1.15	0.797
	Qol-physical	76.4 (2.07)	–	75.0 (2.57)	−1.35 (2.08)	−5.54 to 2.84	0.519
	Qol-psychological	75.8 (1.66)	–	75.8 (1.79)	−0.02 (1.63)	−3.26 to 3.23	0.991
	Qol-social	75.2 (2.30)	–	74.5 (2.29)	−0.66 (1.41)	−3.46 to 2.15	0.642
	Qol-environment	85.2 (1.38)	–	84.1 (1.80)	−1.10 (1.52)	−4.13 to 1.93	0.470
	Qol-overall	70.9 (2.67)	–	68.9 (2.56)	−2.03 (2.25)	−6.50 to 2.44	0.370

a *T2, T3, and T4 assessments of the delayed treatment group were combined with T0, T1, and T2 assessments of the immediate treatment group*.

b *Missing assessments in two patients of the delayed treatment group*.

c *Missing assessments in one patient of the immediate treatment group and five patients of the delayed treatment group*.

d *Comparison (T2–T1) for treatment satisfaction*.

*T0, Baseline; T1, Post-treatment; T2, 3-months follow-up; Mean, Model Estimated Mean; SE, Standard Error of Mean*.

*HADS, Hospital Anxiety and Depression Scale; Qol, WHOQOL-Bref; PDI, Patient Dignity Inventory; FACIT-Sp-12, FACIT – Spiritual Well-Being*.

### Comparison in Study Partners (Family Members and Close Friends)

We finally tested whether Dignity Therapy had any effects on outcome measures in family members/close friends. As displayed in Table 3, we did not find any significant differences between the baseline and 3-months follow-up scores in all measures (HADS, WHOQOL-Bref).

## Discussion

Our main finding was that Dignity Therapy, a short psychotherapeutic intervention to enhance the sense of dignity, was feasible and highly accepted in a sample of patients with early stage dementia and in their family members. Despite SARS-Cov2-Pandemic problems (recruiting stop of more than 2 months) we successfully enrolled enough patients over a period of 2 years and the final participation rate of 38.6% of all screened and invited individuals was almost as high as at least expected and comparable to other studies (19). This is an important finding, because one could expect that the inclusion of patients with early stage dementia in such a study could be too demanding and therefore not feasible. In fact, the most frequent reason for refusal of participation were study related requirements such as filling in questionnaires and to keep appointments. A further reason for non-participation were physical or mental problems (*n* = 6), which, however, was lower than expected in this sample of older participants (mean age = 79.5 years).

Acceptability was high with a drop-out rate of 11.1%, which was significantly lower than expected at worst and also much lower than reported in other psychotherapy studies, ranging between 10 and 70% (35). Importantly, the majority (five out of six participants) of drop-outs was related to the waiting list condition. Additionally, treatment satisfaction was very high, both in patients and study partners. In patients, treatment satisfaction even increased from post-treatment to the 3-months follow-up. In response to open-ended questions, most of the patients reported that the discussion of meaningful events and achievements in their lives as well as the generativity document were the most helpful parts of the intervention.

In terms of the secondary outcomes, we found significant improvements between baseline and 3-months follow-up in almost all measures (HADS, PDI, physical, social, and environment domains of quality of life as well as in spiritual well-being) in the immediate treatment group. This effect was confirmed and even stronger after we pooled the treatment data of the two groups. However, this study was not able to demonstrate a significant group by time effect for any of the outcome measures. This finding was not entirely surprising, because this study was not primarily designed to detect such an effect as argued in our power analysis. Further studies with larger sample sizes and enough power are therefore needed. Furthermore, in our study the effect size for HADS scores between the groups was small (Cohen's d = 0.1), which might be explained by a floor effect: the mean HADS totals scores were very low and with no serious evidence for a clinically relevant depression or anxiety disorder. In the absence of such clinical symptoms, there is little room for improvement. Future studies, therefore, should consider screenings for psychological distress before enrollment to improve the likelihood of detecting differences.

In this study we also invited family members or close friends to accompany the patients during the intervention. The main reason for this setting was that family members or close friends who are known to be distressed by the diagnosis of dementia (36, 37), would benefit from Dignity Therapy. Although study partners were higher distressed and like patients reported high treatment satisfaction, we did not find any effect on psychological burden and quality of life. Probably, such effects would be more visible and detectable in the long-term, when affected patients turn to more severe dementia.

### Strengths and Limitations of Study

This is the first randomized, waitlist controlled study to determine feasibility, acceptability, and potential efficacy of Dignity Therapy in patients with early stage dementia and their family members. The present trial showed that Dignity Therapy was feasible, highly accepted and potential effective. However, because of the primary aim of the study and the accordingly study design this study was underpowered to demonstrate significant differences between the immediate and delayed treatment group in the RCT condition. Further research with larger samples and probably inclusion of only pre-screened patients with moderate psychological distress is needed to better ascertain the efficacy of this intervention among patients with early stage dementia. This study has some other limitations. It had a short-term follow-up and as a potential selection bias included patients and study partners with very low psychological burden. The included study partners were rather heterogeneous and future studies need to examine whether feasibility and acceptance varies among different caregiver constellations. Further, according to the study design using a waiting list control instead of a control group without intervention, there might have been an optimistic expectation in waiting list participants too. Another limitation is that we did not assess fidelity of the intervention systematically, for instance, by analyzing the audio-taped interviews or the transcribed generativity documents. However, all therapists were thoroughly trained and regularly supervised by the study coordinator and first author. Finally, the study coordinator who was responsible for the assessments was not blinded for the randomization. However, in this trial we used exclusively self-report instruments which reduces the probability of a potential detection bias (38).

## Conclusions

Results of this randomized clinical trial demonstrated the feasibility, acceptability, and potential efficacy of Dignity Therapy among patients with early stage dementia. Dignity Therapy might be a promising intervention to enhance dignity among those patients and their family members or close friends. It, therefore, might have the potential to close an existing gap in the holistic treatment of dementia. Further studies are needed in larger and more distressed populations.

## Data Availability Statement

The raw data supporting the conclusions of this article will be made available by the authors, without undue reservation.

## Ethics Statement

The studies involving human participants were reviewed and approved by Cantonal Ethics Committee of Zurich (BASEC-Nr.2018-01097). The patients/participants provided their written informed consent to participate in this study.

## Author Contributions

JJ, HM, PM, TM-H, and SP-K were responsible for the study conception and design. JJ and HM drafted the manuscript. All authors contributed to the acquisition, analysis, or interpretation of data and also provided a critical review, approved the final manuscript and had full access to all the data in the study and take responsibility for the integrity of the data and the accuracy of the data analyses. JJ attests that all listed authors meet authorship criteria and that no others meeting the criteria have been omitted.

## Funding

This work was supported by Porticus (KRS-144045; PCG-155468).

## Conflict of Interest

The authors declare that the research was conducted in the absence of any commercial or financial relationships that could be construed as a potential conflict of interest.

## Publisher's Note

All claims expressed in this article are solely those of the authors and do not necessarily represent those of their affiliated organizations, or those of the publisher, the editors and the reviewers. Any product that may be evaluated in this article, or claim that may be made by its manufacturer, is not guaranteed or endorsed by the publisher.

## References

[1] Prince M Wimo A Guerchet M Ali G Wu Y Prina M. World Alzheimer Report 2015: The Global Impact of Dementia: An Analysis of Prevalence, Incidence, Cost and Trends. Alzheimer's Disease International, London (2015).

[2] Federal Office of Public Health (FOPH). National Dementia Strategy 2014-2017. Bern: FOPH and CMPH (2014). p. 15. Available online at: www.nationaldementiastrategy; http://www.bag.admin.ch/themen/gesundheitspolitik/13916/index.html?lang=en.

[3] Robinson L Tang E Taylor JP. Dementia: timely diagnosis and early intervention. BMJ. (2015) 350:h3029. 10.1136/bmj.h3029 26079686 PMC4468575

[4] World Health Organization WHO. Dementia Fact Sheet.World Health Organization (2020). Available online at: https://www.who.int/news-room/fact-sheets/detail/dementia.

[5] Morris C. A global update on the development of palliative care services. Int J Palliat Nurs. (2011) 17:472, 474, 476. 10.12968/ijpn.2011.17.10.472 22068116

[6] Chochinov HM Cann B Cullihall K Kristjanson L Harlos M McClement SE. Dignity therapy: a feasibility study of elders in long-term care. Palliat Support Care. (2012) 10:3–15. 10.1017/S1478951511000538 22329932

[7] Goddard C Speck P Martin P Hall S. Dignity therapy for older people in care homes: a qualitative study of the views of residents and recipients of 'generativity' documents. J Advanc Nurs. (2013) 69:122–32. 10.1111/j.1365-2648.2012.05999.x 22489609

[8] World Health Organization Alzheimer's Disease International. Dementia - A Public Health Priority. Geneva: World Health Organization (2012). Available online at: https://apps.who.int/iris/bitstream/handle/10665/75263/978?sequence.

[9] World Health Organization. Ensuring a Human Rights-Based Approach for People Living With Dementia. Geneva: World Health Organization (2015). Available online at: http://www.who.int/mental_health/neurology/dementia/en/.

[10] Chochinov HM Hack T Hassard T Kristjanson LJ McClement S Harlos M. Dignity therapy: a novel psychotherapeutic intervention for patients near the end of life. J Clin Oncol. (2005) 23:5520–5. 10.1200/JCO.2005.08.391 16110012

[11] Chochinov HM Kristjanson LJ Breitbart W McClement S Hack TF McClement S. Effect of dignity therapy on distress and end-of-life experience in terminally ill patients: a randomised controlled trial. Lancet Oncol. (2011) 12:753–62. 10.1016/S1470-2045(11)70153-X 21741309 PMC3185066

[12] Bentley B O'Connor M Kane R Breen LJ. Feasibility, acceptability, and potential effectiveness of dignity therapy for people with motor neurone disease. PLoS ONE. (2014) 9:e96888. 10.1371/journal.pone.0096888 24816742 PMC4016138

[13] Johns SA. Translating dignity therapy into practice: effects and lessons learned. Omega. (2013) 67:135–45. 10.2190/OM.67.1-2.p 23977789

[14] Hall S Edmonds P Harding R Chochinov H Higginson IJ. Assessing the feasibility, acceptability and potential effectiveness of Dignity Therapy for people with advanced cancer referred to a hospital-based palliative care team: study protocol. BMC Palliative Care. (2009) 8:5. 10.1186/1472-684X-8-5 19445711 PMC2689205

[15] Houmann LJ Chochinov HM Kristjanson LJ Petersen MA Groenvold M. A prospective evaluation of dignity therapy in advanced cancer patients admitted to palliative care. Palliat Med. (2014) 28:448–58. 10.1177/0269216313514883 24311296

[16] Juliao M Nunes B Barbosa A. Dignity therapy and its effect on the survival of terminally ill Portuguese patients. Psychother Psychosom. (2015) 84:57–8. 10.1159/000366207 25547879

[17] Akechi T Akazawa T Komori Y Morita T Otani H Shinjo T. Dignity therapy: preliminary cross-cultural findings regarding implementation among Japanese advanced cancer patients. Palliat Med. (2012) 26:768–9. 10.1177/0269216312437214 22733965

[18] Fitchett G Emanuel L Handzo G Boyken L Wilkie DJ. Care of the human spirit and the role of dignity therapy: a systematic review of dignity therapy research. BMC Palliative Care. (2015) 14:8. 10.1186/s12904-015-0007-1 25844066 PMC4384229

[19] Hall S Goddard C Opio D Speck P Higginson IJ. Feasibility, acceptability and potential effectiveness of Dignity Therapy for older people in care homes: a phase II randomized controlled trial of a brief palliative care psychotherapy. Palliat Med. (2012) 26:703–12. 10.1177/0269216311418145 21859743

[20] Johnston B Lawton S McCaw C. Living well with dementia: enhancing dignity and quality of life, using a novel intervention, dignity therapy. Int J Older People Nurs. (2015) 11:107–20. 10.1111/opn.12103 26710890

[21] Heggestad AK Slettebo A. How individuals with dementia in nursing homes maintain their dignity through life storytelling - a case study. J Clin Nurs. (2015) 24:2323–30. 10.1111/jocn.12837 25895057

[22] Morris JC. The Clinical Dementia Rating (CDR): current version and scoring rules. Neurology. (1993) 43:2412–14. 10.1212/WNL.43.11.2412-a 8232972

[23] Hawthorne G Sansoni J Hayes L Marosszeky N Sansoni E. Measuring patient satisfaction with health care treatment using the Short Assessment of Patient Satisfaction measure delivered superior and robust satisfaction estimates. J Clin Epidemiol. (2014) 67:527–37. 10.1016/j.jclinepi.2013.12.010 24698296

[24] Stott J Spector A Orrell M Scior K Sweeney J Charlesworth G. Limited validity of the Hospital Anxiety and Depression Scale (HADS) in dementia: evidence from a confirmatory factor analysis. Int J Geriatr Psychiatry. (2017) 32:805–13. 10.1002/gps.4530 27352820

[25] Di Lorenzo R Cabri G Carretti E Galli G Giambalvo N Rioli G. A preliminary study of Patient Dignity Inventory validation among patients hospitalized in an acute psychiatric ward. Neuropsychiatr Dis Treat. (2017) 13:177–90. 10.2147/NDT.S122423 28182110 PMC5279815

[26] Lucas-Carrasco R Skevington SM Gómez-Benito J Rejas J March J. Using the WHOQOL-BREF in persons with dementia: a validation study. Alzheimer Dis Assoc Disord. (2011) 25:345–51. 10.1097/WAD.0b013e31820bc98b 21297426

[27] Hall S Beatty S. Assessing spiritual well-being in residents of nursing homes for older people using the FACIT-Sp-12: a cognitive interviewing study. Qual Life Res. (2014) 23:1701–11. 10.1007/s11136-014-0627-6 24470288

[28] Bjelland I Dahl AA Haug TT Neckelmann D. The validity of the Hospital Anxiety and Depression Scale. An updated literature review. J Psychosom Res. (2002) 52:69–77. 10.1016/S0022-3999(01)00296-3 11832252

[29] Zigmond AS Snaith RP. The hospital anxiety and depression scale. Acta Psychiatr Scand. (1983) 67:361–70. 10.1111/j.1600-0447.1983.tb09716.x 6880820

[30] Wu Y Levis B Sun Y He C Krishnan A Neupane D. Accuracy of the Hospital Anxiety and Depression Scale Depression subscale (HADS-D) to screen for major depression: systematic review and individual participant data meta-analysis. BMJ. (2021) 373:n972. 10.1136/bmj.n972 33972268 PMC8107836

[31] Chochinov HM Hassard T McClement S Hack T Hack T Harlos M. The patient dignity inventory: a novel way of measuring dignity-related distress in palliative care. J Pain Symptom Manage. (2008) 36:559–71. 10.1016/j.jpainsymman.2007.12.018 18579340

[32] Sautier LP Vehling S Mehnert A. Assessment of patients' dignity in cancer care: preliminary psychometrics of the german version of the Patient Dignity Inventory (PDI-G). J Pain Symptom Manage. (2014) 47:181–8. 10.1016/j.jpainsymman.2013.02.023 23830532

[33] Angermeyer MC Kilian R Matschinger H. WHOQOL-100 und WHOQOL-BREF. Göttingen; Bern; Toronto, ON; Seattle, DC: Hogrefe (2000).

[34] Peterman A Fitchett G Brady M Hernandez L Cella D. Measuring spiritual well-being in people with cancer: the functional assessment of chronic illness therapy–Spiritual Well-being Scale (FACIT-Sp). Ann Behav Med. (2002) 24:49–58. 10.1207/S15324796ABM2401_06 12008794

[35] Cuijpers P Quero S Noma H Ciharova M Miguel C Miguel C. Psychotherapies for depression: a network meta-analysis covering efficacy, acceptability and long-term outcomes of all main treatment types. World Psychiatry. (2021) 20:283–93. 10.1002/wps.20860 34002502 PMC8129869

[36] Black W Almeida OP. A systematic review of the association between the Behavioral and Psychological Symptoms of Dementia and burden of care. Int Psychogeriatr. (2004) 16:295–315. 10.1017/S1041610204000468 15559754

[37] Cameron ID Aggar C Robinson AL Kurrle SE. Assessing and helping carers of older people. BMJ. (2011) 343:d5202. 10.1136/bmj.d5202 21930805

[38] Higgins JPT Altman DG Gøtzsche PC Jüni P Moher D Oxman AD. The Cochrane Collaboration's tool for assessing risk of bias in randomised trials. BMJ. (2011) 343:d5928. 10.1136/bmj.d5928 22008217 PMC3196245

