# Supplementary material for: Feasibility, Acceptability, and Preliminary Efficacy of Dignity Therapy in Patients With Early Stage Dementia and Their Family. A Pilot Randomized Controlled Trial
Source: Front Psychiatry. 2021 Dec 24;12:795813. doi: 10.3389/fpsyt.2021.795813 (PMC8740176; doi:10.3389/fpsyt.2021.795813)
Supplement: Supplementary file 1 [file Data_Sheet_1.docx]

**Supplementary appendix**

**Protocol for questions about Dignity Therapy (Chochinov, 2012)**

1. Tell me a little about your life history, particularly the parts that you either remember most or think are the most important? When did you feel most alive?
2. Are there specific things that you would want your family to know about you, and are there particular things you would want them to remember?
3. What are the most important roles you have had in life (e.g., family roles, vocational roles, community-service roles)?
4. What are your most important accomplishments, and what do you feel most proud of?
5. Why were they so important to you and what do you think you accomplished in those roles?
6. Are there particular things that you feel still need to be said to your loved ones or things that you would want to take the time to say once again?
7. What are your hopes and dreams for your loved ones?
8. What have you learned about life that you would want to pass along to others?
9. What advice or words of guidance would you wish to pass along to your son, daughter, husband, wife, parents, or other(s)?
10. Are there words or perhaps even instructions that you would like to offer your family to help prepare them for the future?
11. In creating this permanent record, are there other things that you would like included?

**Dignity Therapy Evaluation Questionnaire (DTEQ)**

Below you find some questions regarding your satisfaction with Dignity Therapy. Please mark or circle one number per line to indicate your response.

|  | **Not at all** | **A little bit** | **Some-**  **what** | **Quite a bit** | **Very much** |
| --- | --- | --- | --- | --- | --- |
| 1. Did you perceive Dignity Therapy as helpful? | 1 | 2 | 3 | 4 | 5 |
| 1. Would you recommend Dignity Therapy to other patients? | 1 | 2 | 3 | 4 | 5 |
| 1. Are you satisfied with your own answers/comments given in/during the therapy? | 1 | 2 | 3 | 4 | 5 |
| 1. Did you feel yourself well supported by the therapist? | 1 | 2 | 3 | 4 | 5 |
| 1. Did you experience Dignity Therapy as meaningful? | 1 | 2 | 3 | 4 | 5 |
| 1. Did you feel treated yourself respectfully during therapy? | 1 | 2 | 3 | 4 | 5 |
| 1. Are you overall satisfied with Dignity Therapy? | 1 | 2 | 3 | 4 | 5 |
| 1. How much was the conversation with the therapist helpful? | 1 | 2 | 3 | 4 | 5 |
| 1. How much was the generativity dokument helpful? | 1 | 2 | 3 | 4 | 5 |
